# Validation of Revised Chinese Version of PD-CRS in Parkinson's Disease Patients

**DOI:** 10.1155/2020/5289136

**Published:** 2020-02-19

**Authors:** Yuyan Tan, Weiguo Liu, Juanjuan Du, Miaomiao Hou, Cuiyu Yu, Yang Liu, Shishuang Cui, Lei Yan, Yizhou Lu, Hong Lv, Lijun Han, Xi Wang, Shengyu Zha, Xiaoguang Luo, Huidong Tang, Shengdi Chen

**Affiliations:** ^1^Department of Neurology and Institute of Neurology, Ruijin Hospital Affiliated to Shanghai Jiao Tong University School of Medicine, Shanghai 200025, China; ^2^Department of Neurology, Affiliated Nanjing Brain Hospital of Nanjing Medical University, Nanjing, Jiangsu 210029, China; ^3^Department of Neurology, Ruijin Hospital North Affiliated to Shanghai Jiao Tong University School of Medicine, Shanghai 201800, China; ^4^Department of Neurology, The First Affiliated Hospital of China Medical University, Shenyang 110001, China; ^5^Department of Neurology, Shenzhen People's Hospital, Second Clinical Medical College of Jinan University, Shenzhen 518020, China

## Abstract

There is a high prevalence of mild cognitive impairment (MCI) and dementia in Parkinson's disease (PD) patients, but a Chinese version of cognitive rating scale that is specific and sensitive to PD patients is still lacking. The aims of this study are to test the reliability and validity of a Chinese version of Parkinson's disease-cognitive rating scale (PD-CRS), establish cutoff scores for diagnosis of Parkinson's disease dementia (PDD) and PD with mild cognitive impairment (PD-MCI), explore cognitive profiles of PD-MCI and PDD, and find cognitive deficits suggesting a transition from PD-MCI to PDD. PD-CRS was revised based on the culture background of Chinese people. Ninety-two PD patients were recruited in three PD centers and were classified into PD with normal cognitive function (PD-NC), PD-MCI, and PDD subgroups according to the cognitive rating scale (CDR). Those PD patients underwent PD-CRS blind assessment by a separate neurologist. The PD-CRS showed a high internal consistency (Cronbach's Alpha = 0.840). Intraclass Correlation coefficient (ICC) of test-retest reliability reached 0.906 (95% CI 0.860–0.935, *p* < 0.001). ICC of inter-rater reliability was 0.899 (95% CI 0.848–0.933, *p* < 0.001). PD-CRS had fair concurrent validity with MDRS (ICC = 0.731, 95% CI 0.602–0.816). All the frontal-subcortical items showed significant decrease in PD-MCI compared with the PD-NC group (*p* ≤ 0.001), but the instrument cortical items did not (confrontation naming *p*=0.717, copying a clock *p*=0.620). All the frontal-subcortical and instrumental-cortical functions showed significant decline in PDD compared with the PD-NC group (*p* ≤ 0.001). The cutoff value for diagnosis of PD-MCI is 80.5 with the sensitivity of 75.7% and the specificity of 75.0%, and for diagnosis of PDD is 73.5 with the sensitivity of 89.2% and the specificity of 98.9%. Revised Chinese version of PD-CRS is a reliable, acceptable, valid, and useful neuropsychological battery for assessing cognition in PD patients.

## 1. Introduction

Cognitive impairment is common in Parkinson's disease (PD), even in its early stages. Mild cognitive impairment (MCI) may be identified in approximately 25% of newly diagnosed patients [1], and those PD patients are at a higher risk of developing dementia compared with normal cognition PD patients [2–4]. Parkinson's disease dementia (PDD) has a cumulative prevalence up to 75–90% of those with a disease duration of 10 years or more [5]. Cognitive impairment in PD patients includes attention deficits, executive dysfunction, visuospatial defects, free-recall memory problems, confrontation naming difficulties, as well as encoding deficits [6–8].

Diagnosis of PDD largely relies on neuropsychological measurements and evaluation. Four neuropsychological evaluation tools have been designed specifically for PD patients so far. Minimental Parkinson (MMP) and Parkinson Neuropsychometric Dementia Assessment (PANDA) are short screen tests for cognitive impairment in PD patients, but lack extensive clinimetric evaluation [9–11]. Scale for Outcomes of Parkinson's Disease-Cognition (SCOPA-COG) is a reliable and valid instrument for assessing “frontal-subcortical” function, but the “instrumental-cortical” function is missing [12], which has been identified in approximately 15–20% of PD patients [6]. Parkinson's disease-cognitive rating scale (PD-CRS), designed by Dr. Kulisevsky, is a comprehensive, reliable, and valid instrument for assessing both “frontal-subcortical” functions (sustained attention, working memory, alternating and action verbal fluencies, clock drawing, and immediate and delayed free-recall verbal memory) and “instrumental-cortical” functions (confrontation naming, copying a clock) [13–15]. China has over 2 million PD patients, but cognitive impairment is substantially underestimated because of the lack of a Chinese version neuropsychological evaluation tool specific for PD patients. The aims of the present study are to test the reliability and validity of the Chinese version PD-CRS; establish cutoff scores for diagnosis of PDD and PD-MCI; explore cognitive profiles of PD-MCI and PDD; and find cognitive deficits suggesting a transition from PD-MCI to PDD.

## 2. Materials and Methods

### 2.1. Revised Chinese Version of PD-CRS

China has different culture and language systems from western countries. Therefore, three steps were executed to make ensure that the PD-CRS was adapted to Chinese people. First, the English version of PD-CRS was translated to a provisional Chinese version and was examined in a consensus meeting. Second, the provisional Chinese version of PD-CRS was administered to 15 Chinese healthy volunteers with age ranging from 60–85 and with 6 or more years of education. The preliminary test showed that senior Chinese people were not familiar with some of the pictures in the picture naming section, such as “jingle bell,” “guitar,” “berry,” and “stool.” The Spring Festival is the traditional festival in China, which is similar to the Christmas day in the West. It is a tradition to hang lanterns at the Spring Festival which is akin to hanging jingle bells on Christmas day. Thus, using “lantern” instead of “jingle bell” kept the difficulty level of naming. We made four modifications in the confrontation naming part: “lantern” replaced “jingle bell,” “erhu” replaced “guitar,” “strawberry” replaced “berry,” and “chair” replaced “stool.” All experts approved of these modifications in the picture-naming section in the consensus meeting. We also found that most of these senior Chinese people did not know English letters. We made the following changes to the “sustained attention,” “working memory,” and “alternating verbal fluency,” with the help from Dr. Kulisevsky, the author of PD-CRS. The original instructions in the “sustained attention” section are to read an ascending series of letters and numbers to the subject, asking the subject to say how many letters are there in the series. In the revised Chinese version, the instructions are to read an ascending series of numbers to the subject, asking the subject to say how many odd numbers are there in the series. Thus, in both the original and revised instruction, the subjects need to memorize what they heard and be able to operate classification at the same time. The original instructions in the “working memory” section is to read aloud a randomized list of numbers and letters ranging in length from 2 to 6 letters and numbers. After each series, the subject is asked to repeat the numbers first, and then the letters. In the revised Chinese version, the instructions are to read aloud a randomized list of numbers in length from 2–6 numbers. After each series, the subject is asked to repeat the numbers backward. The revised method is similar to the backward digit span test and tests the subject's working memory. The original instructions in the “alternating verbal fluency” section asks the subject to generate as many different words as possible by alternating between words beginning with the letter “S” and articles of clothing for a 60-second duration. In the revised Chinese version, the instructions are to ask the subject to make as many different phrases as possible by alternating between providing words starting with the written form of Chinese character “发” pronounced as “fa” and articles of clothing for a 60-second duration. Third, after all modifications were completed, the newly revised Chinese version of PD-CRS was finally approved in a consensus meeting. The new version was then retested in 15 Chinese healthy volunteers. All the participants and examiners had good understanding and comprehensibility of the instructions. The revised Chinese version of PD-CRS was attached as supplementary material.

### 2.2. Subjects

A cohort of 92 PD patients were recruited from 3 centers, including the Neurology Department of Ruijin Hospital affiliated to Shanghai Jiao Tong University School of Medicine; Neurology Department of Nanjing Brain Hospital affiliated to Nanjing Medical University; and Neurology Department of the First Affiliated Hospital of China Medical University. The inclusion criteria for the enrollment were diagnosis of idiopathic PD according to the UK Brain Bank, ages of 60–85 years, and 6 or more years of education. The exclusion criteria were other neurological diseases, such as stroke, epilepsy, tumor, brain trauma (history and cranial MRI), and abnormalities on brain CT or MRI in the past 12 months; nutritional and metabolic abnormalities (folic acid or vitamin B12 or vitamin B1 deficiency); psychiatric problems for which who now or used to have psychiatric medicine dependence; serious sleep disorder; history of surgery under general anesthesia within the last year; evidence of physical illness; hearing or vision loss; and severe cardiac or respiratory disorders. This study protocol was approved by the Ethics Committee of Ruijin Hospital affiliated to Shanghai Jiao Tong University School of Medicine. Written informed consent was obtained from all participants in the study as well.

### 2.3. Assessments

For baseline, the collection of demographic and clinical data included age, gender, education, disease duration, past disease history (diabetes, hypertension, coronary heart disease, and cerebrovascular disease), current medications converted to levodopa equivalent daily dose (LEDD), history of smoking or alcohol consumption, the Unified Parkinson's Disease Rating Scale part III (UPDRS-III), Beck Depression Inventory (BDI), and PD-CRS. For the second visit, the same neurologist evaluated the same patient with PD-CRS after 2 weeks. For the third visit, another neurologist evaluated the same patient with PD-CRS, Mattis Dementia Rating Scale (MDRS) and Clinical Dementia Rating (CDR) in an interval of 6 ± 2 weeks from the second visit. Based on CDR, the PD patients were divided into PD-NC, PD-MCI, and PDD subgroups; CDR = 0 in the PD-NC group, CDR = 0.5 in the PD-MCI group, and CDR ≥1 in the PDD group.

### 2.4. Statistical Analysis

All continuous demographic and clinical data were presented as mean ± SD and compared by Analysis Of Variance (ANOVA) or Kruskal–Wallis test. All categorical variables were presented as numbers and estimated by Chi-squared test. Normality of distribution was evaluated by Kolmogorov–Smirnov (K–S) test initially. Test-retest reliability and inter-rater reliability were assessed by intraclass correlation coefficients (ICCs). The ICC is equal to the degree of individual variation divided by the total variability, so the value is between 0 and 1. Landis and Koch recommend ICC should be more than 0.80; 0.61–0.80 classified as good; 0.41–0.60 as fair, 0.11–0.40 as low, and 0.1 or less as no consistency. Internal consistency reliability was evaluated by the Cronbach's alpha coefficient (≥0.80 was considered acceptable) and the corrected item-total correlation (≥0.40 was considered acceptable). Acceptability rating was determined as acceptable for each PD-CRS item if there was <5% of missing data rates and <15% of the floor/ceiling effects (floor: the proportion of patients with the minimum possible score; ceiling: the proportion of patients with the maximum possible score). Receiver operator characteristic (ROC) curves were generated to identify the discriminative power of PD-CRS for diagnosing PD-MCI and PDD. Sensitivity, specificity, positive predictive value (PPV), negative predictive value (NPV), positive likelihood ratios (LR+), and negative likelihood ratios (LR−) were calculated. The appropriate cutoff point was chosen according to the maximum combined sensitivity and specificity. All tests were two-sided, and the results were considered statistically significant at *p* < 0.05. Statistical analysis was performed using SPSS 20.0.

## 3. Results

### 3.1. Demographic and Clinical Data

The demographic and clinical data were presented in Table 1. Of the 92 PD patients, 37 were classified into the PD-NC group, 44 into the PD-MCI group, and 11 into the PDD group. The distributions of age, gender, education, history of smoking, alcohol consumption, diabetes, hypertension, coronary heart disease, and cerebrovascular disease were similar between the groups (*p* > 0.05). There were significant differences in disease duration, H–Y staging, LEDD, UPDRS-III, BDI, and MDRS scores among the three groups (*p* < 0.05). PDD patients have longer disease duration, higher scores of UPDRS-III and BDI, and lower scores of MDRS.

### 3.2. Reliability

Cronbach's alpha was used to measure internal consistency of the PD-CRS scale. The PD-CRS showed a high internal consistency among all items in this scale (Cronbach's Alpha = 0.840). Correction item − total correlation ranged from 0.452 (confrontation naming) to 0.730 (alternating verbal fluencies) (Table 2). No item improved Cronbach's alpha (0.840) if removed. As for the test-retest reliability, the intraclass correlation coefficient (ICC) for each item score of the PD-CRS is presented in Table 2. ICC of the total score of PD-CRS reached 0.906 (95% CI 0.860–0.935, *p* < 0.001), which indicated high test-retest reliability. ICC of each item ranged from 0.691 to 0.825 (*p* < 0.001). For inter-rater reliability (Table 2), the ICC of the total PD-CRS score was 0.899 (95% CI 0.848–0.933, *p* < 0.001), and the ICC of each item ranged from 0.592 to 0.826 (*p* < 0.001). These results indicated that the revised Chinese version of PD-CRS has good internal consistency, test-retest reliability, and inter-rater reliability according to the criteria mentioned in Section 2.4.

### 3.3. Acceptability

Ceiling effect (>15% of the respondents with the highest possible score) and floor effect (>15% of the respondents with the lowest possible score) were analyzed. Nonfloor effects were observed for the total, subcortical, and cortical scores of the PD-CRS when analyzed in all PD patients, specifically PD-NC and PD-MCI subgroup (Table 3, Supplementary Tables [Supplementary-material supplementary-material-1] and [Supplementary-material supplementary-material-1]). But in the PDD subgroup, items of immediate free-recall verbal memory, confrontation naming, sustained attention, working memory, alternating verbal fluencies, and delayed free-recall verbal memory showed floor effects (Supplementary [Supplementary-material supplementary-material-1]), indicating that those cognitive functions were severely and commonly impaired in PDD patients. The ceiling effect was observed in confrontation naming (15.2%), clock drawing (32.6%), and copying a clock (72.8%) (Table 3) when analyzed in whole PD patients, and more items (confrontation naming 21.6%, sustained attention 21.6%, clock drawing 54.1% and copying a clock 86.5%) showed ceiling effects in the PD-NC subgroup (Supplementary [Supplementary-material supplementary-material-1]), whereas only copying a clock showed the ceiling effect (20.5%) in the PD-MCI subgroup (Supplementary [Supplementary-material supplementary-material-1]), indicating that the ceiling effects were mainly due to the PD-NC group.

### 3.4. Concurrent and Discriminative Validity

Concurrent validity was analyzed in total PD-CRS scores with MDRS scores, as well as subscales of PD-CRS with corresponding parts of MDRS (Table 4). PD-CRS showed fair concurrent validity with the MDRS scores (ICC = 0.731, 95% CI 0.602–0.816). The concurrent validity of PD-CRS-working memory with the digit span forward and backward subtest (A) of MDRS (ICC = 0.408, 95% CI 0.223–0.577); alternating verbal fluencies with initiation-preservation subscale (E) of MDRS (ICC = 0.470, 95% CI 0.261–0.625); and delayed free-recall verbal memory with free memory (AF + AG) of MDRS (ICC = 0.638, 95% CI 0.503–0.749) are shown in Table 4. These results show that the concurrent validity of subscales of PD-CRS with corresponding parts of MDRS only reach the fair scope of ICC (0.41–0.60) according to the criteria recommended by Landis and Koch. We think it is due to the different difficulty degrees of these two scales. For example, the working memory subscale of PD-CRS is a randomized list of numbers in length from 2–6 numbers and asking the subject to repeat the numbers backward. In digit span test of MDRS, it is a randomized list of numbers in length 2–4.

For discriminative validity, significant differences were observed in total PD-CRS, frontal-subcortical functions, and instrumental-cortical functions, and each PD-CRS item scores among PD-NC, PD-MCI, and PDD groups (*p* < 0.001) (Table 5). PD-MCI and PDD patients perform differently when compared with PD-NC patients. The frontal-subcortical items showed a significant decrease in the PD-MCI subgroup compared with the PD-NC subgroup (*p* < 0.05), but the instrument cortical items did not (confrontation naming *p*=0.717 and copying a clock *p*=0.620), which means that the cortical functions are relatively intact in PD-MCI patients (instrumental-cortical functions, *p*=0.203), but the subcortical functions are impaired (frontal-subcortical functions, *p* < 0.001). All the frontal-subcortical and instrumental-cortical functions showed significant decline in the PDD subgroup compared with the PD-NC subgroup, which indicates that PDD patients had global cognitive impairment. PDD patients had lowered scores in cortical functions than PD-MCI patients (instrumental-cortical functions 21.64 ± 4.523 vs. 26.66 ± 2.272, *p*=0.004; confrontation naming 14.91 ± 2.809 vs. 17.09 ± 2.351, *p*=0.051; copying a clock 6.73 ± 3.495 vs. 9.57 ± 0.818, *p* < 0.001), but there was no significant difference between PD-MCI and PDD in some of the subcortical functions, such as working memory (*p*=0.168), clock drawing (*p*=0.143), and alternating verbal fluencies (*p*=0.069).

**Figure 1:**
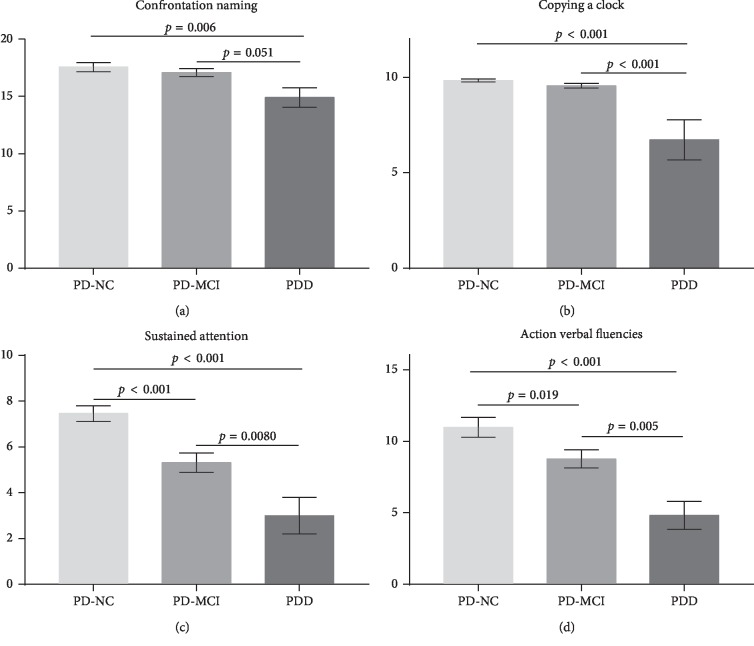
Comparative progression of impairment of “frontal-subcortical functions” and “instrumental-cortical functions” in PD-NC, PD-MCI, and PDD subgroups (mean ± SE). Cortical functions (confrontation naming and copying a clock) are relatively normal in PD-MCI, but had abrupt decline in the PDD group (a and b). Sustained attention and action verbal fluencies were used as examples to show marked decline of subcortical functions in both PD-MCI and PDD (c and d).

Comparative progression of impairment of “frontal-subcortical functions” and “instrumental-cortical functions” showed that those cortical functions (confrontation naming, copying a clock) are relatively normal in PD-MCI, but had abrupt decline in the PDD group (Figures 1(a)1(b)). Subcortical functions had marked decline in both PD-MCI and PDD. Sustained attention and action verbal fluencies were listed as examples (Figures 1(c) and 1(d)). These results indicated that PD-MCI patients and PDD patients have different cognitive impairment profiles and patterns. The worse performance in cortical functions of PDD patients than PD-MCI patients showed a pattern of cognitive impairment transition from PD-MCI to PDD.

### 3.5. Discriminative Power of PD-CRS for Diagnosing PD-MCI and PDD

**Figure 2:**
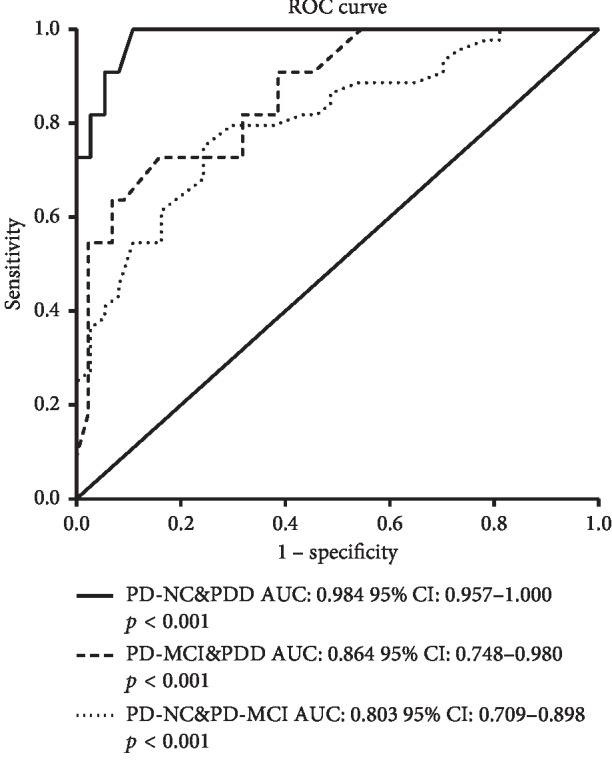
Discriminative power of PD-CRS for diagnosing PD-MCI and PDD. AUC for differentiating PD-MCI is 0.803, 95% CI: 0.709–0.898, *p* < 0.001. AUC for detecting PDD is 0.984, 95% CI: 0.957–1.000, *p* < 0.001.

ROC curve indicated that a PD-CRS total score of 80.5 raised the maximum cutoff accuracy for detecting PD-MCI (AUC: 0.803, 95% CI: 0.709–0.898, *p* < 0.001, sensitivity 75.7%, specificity 75.0%, PPV 75.2%, and NPV 75.5%) (Figure 2, Table 6). The PD-CRS total score of 73.5 is the maximum accuracy cutoff for detecting PDD (AUC: 0.984, 95% CI: 0.957–1.000, *p* < 0.001, sensitivity 89.2%, specificity 98.9%, PPV 98.8%, and NPV 90.1%) (Figure 2, Table 6).

## 4. Discussion

This Chinese version of PD-CRS was revised based on the culture background of Chinese people. Our results showed that the revised Chinese version of PD-CRS is a reliable, acceptable, valid, and useful neuropsychological battery that could accurately diagnose PDD as proven in previous reports [13, 14]. The PD-CRS showed a high internal consistency, test-retest reliability, and inter-rater reliability. The Chinese version of PD-CRS showed fair concurrent validity with the Chinese version of MDRS. No floor effects were observed in the total score of PD-CRS and individual items in whole PD patients, PD-NC, and PD-MCI subgroups; but items of immediate free-recall verbal memory, confrontation naming, sustained attention, working memory, alternating verbal fluencies, and delayed free-recall verbal memory showed floor effects in the PDD group, indicating that PDD patients were commonly and severely impaired in these functions. Ceiling scores were found in confrontation naming, clock drawing, and copying a clock in PD patients analyzed as a whole, but the ceiling scores were mainly distributed among PD-NC patients, and those items were still able to discriminate cognitive impairments in PD-MCI and PDD patients.

There is a spectrum of cognitive dysfunction, ranging from MCI to dementia in PD patients [16]. Executive dysfunction, impaired verbal fluency, visuospatial deficits, as well as encoding memory dysfunction are cognitive profiles of PD-MCI [6–8]. At later stages, both subcortical and cortical functions might be impaired [17]. PD-MCI subjects differed from PD-NC patients in all frontal-subcortical items, whereas the two instrumental-cortical functions items were relatively intact in PD-MCI patients, but were selectively impaired in PDD patients. These results showed different cognitive impairment patterns between PD-MCI and PDD patients. The cutoff value for diagnosis of PD-MCI is 80.5 with the sensitivity of 75.7% and the specificity of 75.0%. PD-MCI subjects are at a higher risk to develop dementia compared with normal cognition PD patients; thus, the discriminant ability to diagnose PD-MCI by the PD-CRS suggests that this scale may be a good instrument for screening purposes. PD-CRS could accurately diagnose PDD, and the cutoff value for diagnosis of PDD is 73.5 with the sensitivity of 89.2% and the specificity of 98.9%. PDD patients showed a significant difference with PD-NC in all subcortical and cortical items, indicating PDD patients had global cognitive impairments.

There were two limitations in the present study. First, we have small sample of PDD patients which might cause bias to some results, such as the high level of floor effects in PDD subgroups. Second, PD patients with high BDI scores which might act as a confounding factor for cognitive function test were not excluded. There were 7 out of 44 PD-MCI patients (15.91%) and 4 out of 11 PDD patients (36.36%) who had BDI scores ≥20. The cognitive function was analyzed between BDI <20 and BDI ≥20 in PD-MCI and PDD subgroups separately. The results showed that PD-CRS total score and each item score have no significant difference between BDI <20 and BDI ≥20 scores in both PD-MCI and PDD subgroups (Supplementary Tables [Supplementary-material supplementary-material-1] and [Supplementary-material supplementary-material-1]). A link between mood symptoms and cognitive impairment in PD has been found, but studies have been inconsistent regarding the relationship between mood symptoms and cognitive function. Ng et al. did not find significant correlation between early depression and cognitive function in both baseline and follow-up tests [18]. Jones et al. reported that depressive symptoms may be a harbinger for future cognitive decline among PD patients [19]. Petkus et al. found that poorer cognitive performance, across all cognitive domains, was a risk factor for increased symptoms of anxiety and depression [20]. In the present study, although we did not find significant difference of cognitive function between different BDI scores subgroups, it would be better to match BDI scores between subgroups to exclude the potential effects of depression on cognitive test.

## 5. Conclusion

Overall, our results showed that the Chinese version of PD-CRS is an applicable and valid tool for assessing cognition in PD patients. It is sensitive in detecting the cognitive impairment transition from predominantly subcortical impairments in PD-MCI patients to global cognitive decline in PDD patients.

## Figures and Tables

**Table 1:** Demographic and clinical characteristics between PD-NC, PD-MCI, and PDD groups.

	PD-NC	PD-MCI	PDD	*p*
*N*	37	44	11	—
Age	68.08 ± 6.202	69.82 ± 6.366	71.27 ± 4.563	0.237^a^
Male (%)	25 (67.6%)	34 (77.3%)	7 (63.6%)	0.511^b^
Education (year)	12.35 ± 2.879	11.63 ± 3.441	10.73 ± 2.284	0.225^c^
Disease duration (year)	5.32 ± 5.716	5.18 ± 3.598	7.82 ± 3.401	0.033^c^
H–Y staging	1.70 ± 0.6714	1.90 ± 0.6522	2.45 ± 0.650	0.009^c^
Smoke (−)	34 (91.9%)	38 (86.4%)	10 (90.9%)	0.712^b^
Alcohol (−)	34 (91.9%)	39 (88.6%)	9 (81.8%)	0.656^b^
Diabetes (−)	32 (86.5%)	39 (88.6%)	9 (81.8%)	0.838^b^
Hypertension (−)	27 (73.0%)	29 (65.9%)	7 (63.6%)	0.739^b^
Coronary heart disease (−)	31 (83.8%)	36 (81.8%)	11 (100%)	0.139^b^
Cerebrovascular disease (−)	33 (89.2%)	39 (88.6%)	10 (90.0%)	0.976^b^
Levodopa (+)	25 (67.6%)	36% (81.8%)	10 (90.0%)	0.151^b^
Dopamine agonists (+)	22 (59.5%)	20 (45.5%)	5 (45.5%)	0.418^b^
COMT inhibitor (+)	3 (8.1%)	10 (22.7%)	0 (0%)	0.061^b^
MAO-B inhibitor (+)	13 (35.1%)	8 (18.2%)	1 (9.1%)	0.090^b^
Anticholinergic (+)	0 (0%)	2 (4.5%)	1 (9.1%)	0.177^b^
Amantadine (+)	3 (8.1%)	7 (15.9%)	2 (18.2%)	0.488^b^
LEDD (mg/d)	323.97 ± 249.571	430.73 ± 287.325	540.91 ± 301.719	0.038^a^
UPDRS-III	12.89 ± 8.906	20.48 ± 13.473	26.00 ± 11.773	0.001^c^
BDI	5.68 ± 4.295	11.45 ± 8.019	16.45 ± 10.727	<0.001^c^
MDRS	138.16 ± 6.265	131.43 ± 9.260	114.27 ± 15.755	<0.001^c^

^a^One-way analysis of variance (ANOVA); ^b^Chi-square test; ^c^Kruskal–Wallis test. PD: Parkinson's disease; PD-NC: PD patients with normal cognition; PD-MCI: PD patients with mild cognitive impairment; PDD: PD patients with dementia; H–Y staging: Hoehn and Yahr staging; COMT inhibitor: catechol O-methyltransferase inhibitor; MAO-B inhibitor: monoamine oxidase-B inhibitor; LEDD: levodopa equivalent daily dose; UPDRS-III: the Unified Parkinson's Disease Rating Scale part III; BDI: Beck Depression Inventory; MDRS: Mattis Dementia Rating Scale.

**Table 2:** Reliability and internal consistency for both total and individual item scores of PD-CRS.

Subscale	Test-retest reliability		Inter-rater reliability		Internal consistency	
	ICC (95% CI)	*p*	ICC (95% CI)	*p*	Corrected item − total correlation	Cronbach's alpha if the item is deleted
Immediate free-recall verbal memory	0.817 (0.747–0.867)	<0.001	0.692 (0.580–0.782)	<0.001	0.705	0.830
Confrontation naming	0.717 (0.554–0.844)	<0.001	0.709 (0.562–0.807)	<0.001	0.452	0.837
Sustained attention	0.810 (0.743–0.867)	<0.001	0.775 (0.667–0.857)	<0.001	0.704	0.829
Working memory	0.706 (0.571–0.808)	<0.001	0.650 (0.521–0.759)	<0.001	0.602	0.835
Clock drawing	0.728 (0.506–0.851)	<0.001	0.675 (0.449–0.822)	<0.001	0.629	0.834
Copying a clock	0.814 (0.423–0.925)	<0.001	0.826 (0.429–0.922)	<0.001	0.566	0.838
Delayed free-recall verbal memory	0.825 (0.743–0.894)	<0.001	0.748 (0.614–0.845)	<0.001	0.665	0.829
Alternating verbal fluencies	0.727 (0.608–0.815)	<0.001	0.592 (0.471–0.693)	<0.001	0.730	0.822
Action verbal fluencies	0.691 (0.562–0.796)	<0.001	0.720 (0.596–0.821)	<0.001	0.711	0.821
Frontal-subcortical functions	0.911 (0.865–0.939)	<0.001	0.893 (0.841–0.929)	<0.001	0.977	0.787
Instrumental-cortical functions	0.780 (0.632–0.870)	<0.001	0.789 (0.655–0.872)	<0.001	0.645	0.829
PD-CRS	0.906 (0.860–0.935)	<0.001	0.899 (0.848–0.933)	<0.001	Cronbach's alpha = 0.840	

PD-CRS: Parkinson's disease-cognitive rating scale; ICC: intraclass correlation coefficients; CI: confidence interval.

**Table 3:** Acceptability of PD-CRS.

Item	Mean ± SD	Min-max	Skewness	Kurtosis	Floor effect (%)	Ceiling effect (%)
Immediate free-recall verbal memory	7.32 ± 2.693	0–12	−0.154	−0.653	1.1	6.5
Confrontation naming	17.02 ± 2.580	10–20	−1.131	0.840	4.3	15.2
Sustained attention	5.90 ± 2.894	0–10	−0.357	−0.822	4.3	10.8
Working memory	5.34 ± 2.190	0–10	0.481	−0.177	1.1	5.4
Clock drawing	8.00 ± 2.335	0–10	−1.467	1.812	1.1	32.6
Copying a clock	9.34 ± 1.639	0–10	−4.018	18.591	1.1	72.8
Delayed free-recall verbal memory	5.72 ± 3.068	0–12	−0.124	−0.829	6.5	1.1
Alternating verbal fluencies	7.50 ± 4.040	0–16	−0.191	−0.438	6.5	2.2
Action verbal fluencies	9.18 ± 4.501	0–24	0.417	0.300	2.2	1.1
Frontal-subcortical functions	48.96 ± 15.777	11–82	−0.382	−0.357	2.2	1.1
Instrumental-cortical functions	26.36 ± 3.274	15–30	−1.441	2.082	1.1	13.0
PD-CRS total score	75.32 ± 17.818	30–109	−0.533	−0.092	1.1	1.1

PD-CRS: Parkinson's disease-cognitive rating scale; SD: standard deviation.

**Table 4:** Validity of PD-CRS.

	MDRS		
	ICC	95% CI	*p*
PD-CRS total score	0.731	[0.602, 0.816]	<0.001
Working memory vs. MDRS (A)	0.408	[0.223, 0.577]	<0.001
Alternating verbal fluencies vs. MDRS (E)	0.470	[0.261, 0.625]	<0.001
Delayed free-recall verbal memory vs. MDRS (AF + AG)	0.638	[0.503, 0.749]	<0.001

PD-CRS: Parkinson's disease-cognitive rating scale; MDRS: Mattis Dementia Rating Scale; ICC: intraclass correlation coefficients; CI: confidence interval.

**Table 5:** Comparisons of PD-CRS between PD-NC, PD-MCI, and PDD groups.

	PD-NC	PD-MCI	PDD	*p*	PD-NC vs. PD-MCI^c^	PD-NC vs. PDD^c^	PD-MCI vs. PDD^c^
Immediate free-recall verbal memory	8.81 ± 2.132	6.70 ± 2.681	4.73 ± 1.191	<0.001^a^	<0.001	<0.001	0.014
Confrontation naming	17.57 ± 2.523	17.09 ± 2.351	14.91 ± 2.809	0.008^b^	0.717	0.006	0.051
Sustained attention	7.46 ± 2.116	5.32 ± 2.785	3.00 ± 2.646	<0.001^a^	<0.001	<0.001	0.008
Working memory	6.46 ± 2.445	4.82 ± 1.618	3.64 ± 1.362	<0.001^b^	0.012	0.001	0.168
Clock drawing	9.05 ± 1.311	7.82 ± 2.026	5.18 ± 3.573	<0.001^b^	0.007	<0.001	0.143
Copying a clock	9.84 ± 0.442	9.57 ± 0.818	6.73 ± 3.495	<0.001^b^	0.620	<0.001	<0.001
Delayed free-recall verbal memory	7.43 ± 2.714	5.11 ± 2.572	2.36 ± 2.420	<0.001^a^	<0.001	<0.001	0.002
Alternating verbal fluencies	9.70 ± 3.566	6.64 ± 3.577	3.55 ± 3.045	<0.001^b^	0.004	<0.001	0.069
Action verbal fluencies	10.97 ± 4.213	8.77 ± 4.220	4.82 ± 3.219	<0.001^a^	0.019	<0.001	0.005
Frontal-subcortical functions	59.89 ± 10.448	45.18 ± 12.901	27.27 ± 11.577	<0.001^a^	<0.001	<0.001	<0.001
Instrumental-cortical functions	27.41 ± 2.682	26.66 ± 2.272	21.64 ± 4.523	<0.001^b^	0.203	<0.001	0.004
PD-CRS total score	87.30 ± 11.244	71.84 ± 14.144	48.91 ± 14.916	<0.001^a^	<0.001	<0.001	<0.001

PD-CRS: Parkinson's disease-cognitive rating scale; PD: Parkinson's disease; PD-NC: PD patients with normal cognition; PD-MCI: PD patients with mild cognitive impairment; PDD: PD patients with dementia. ^a^One-way analysis of variance (ANOVA); ^b^Kruskal–Wallis test; ^c^Bonferroni test.

**Table 6:** Accuracy measures of PD-CRS.

Scale version	Cutoff	Sensitivity (%)	Specificity (%)	PPV (%)	NPV (%)	LR (+)	LR (−)
PD-NC/PD-MCI (AUC 0.803)	78.5	78.4	65.9	69.7	75.3	2.29	0.33
	**80.5**	**75.7**	**75.0**	**75.2**	**75.5**	**3.03**	**0.32**
	81.5	73.0	77.3	76.3	74.1	3.22	0.35

PD-MCI/PDD (AUC 0.864)	54.5	90.9	63.6	71.4	87.5	2.49	0.14
	**57.5**	**84.1**	**72.7**	**75.5**	**82.1**	**3.08**	**0.22**
	58.5	77.3	72.7	73.9	76.2	2.83	0.31

PD-NC/PDD (AUC 0.984)	69.0	94.6	90.9	91.2	94.4	10.39	0.06
	**73.5**	**89.2**	**98.9**	**98.8**	**90.1**	**81.09**	**0.11**
	75.0	86.5	99.6	99.5	88.1	216.25	0.14

PD-CRS: Parkinson's disease-cognitive rating scale; SD: standard deviation; PD: Parkinson's disease; PD-NC: PD patients with normal cognition; PD-MCI: PD patients with mild cognitive impairment; PDD: PD patients with dementia; AUC: area under the curve; PPV: positive predictive value; NPV: negative predictive value; LR+: positive likelihood ratio; LR−: negative likelihood ratio.

## Data Availability

The data used to support the findings of this study are available from the corresponding author upon request.

## Supplementary Materials

Supplementary Materials Supplementary Table 1: acceptability of PD-CRS in NC; Supplementary Table 2: acceptability of PD-CRS in PD-MCI; Supplementary Table 3: acceptability of PD-CRS in PDD; Supplementary Table 4: cognitive function comparison between BDI <20 and ≥ 20 in PD-MCI; Supplementary Table 5: cognitive function comparison between BDI <20 and ≥ 20 in PDD.
