# Supplementary material for: Validation of Revised Chinese Version of PD-CRS in Parkinson's Disease Patients
Source: Parkinsons Dis. 2020 Feb 19;2020:5289136. doi: 10.1155/2020/5289136 (PMC7049872; doi:10.1155/2020/5289136)
Supplement: Supplementary Materials — Supplementary Table 1: acceptability of PD-CRS in NC; Supplementary Table 2: acceptability of PD-CRS in PD-MCI; Supplementary Table 3: acceptability of PD-CRS in PDD; Supplementary Table 4: cognitive function comparison between BDI <20 and ≥ 20 in PD-MCI; Supplementary Table 5: cognitive function comparison between BDI <20 and ≥ 20 in PDD. [file 5289136.f1.zip › mat.5289136.v2.docx]

Supplement-Table1 Acceptability of PD-CRS in NC

| Item | Mean±SD | Min-Max | skewness | kurtosis | Floor effect (%) | Ceiling effect（%） |
| --- | --- | --- | --- | --- | --- | --- |
| Immediate free-recall verbal memory | 8.81±2.132 | 5-12 | -0.193 | -0.832 | 8.1 | 13.5 |
| Confrontation naming | 17.57±2.523 | 10-20 | -1.642 | 2.838 | 5.4 | 21.6 |
| Sustained attention | 7.46±2.116 | 1-10 | -0.740 | 0.711 | 2.7 | 21.6 |
| Working memory | 6.46±2.445 | 2-10 | -0.111 | -1.171 | 5.4 | 10.8 |
| Clock drawing | 9.05±1.311 | 6-10 | -1.277 | 0.443 | 8.1 | 54.1 |
| Copying a clock | 9.84±.442 | 8-10 | -2.868 | 8.277 | 2.7 | 86.5 |
| Delayed free-recall verbal memory | 7.43±2.714 | 1-12 | -0.477 | -0.510 | 2.7 | 2.7 |
| Alternating verbal fluencies | 9.70±3.566 | 1-16 | -0.131 | -0.139 | 2.7 | 5.4 |
| Action verbal fluencies | 10.97±4.213 | 4-24 | 0.769 | 1.189 | 2.7 | 2.7 |
| Frontal-subcortical functions | 59.89±10.448 | 37-82 | -0.052 | -0.157 | 2.7 | 2.7 |
| Instrumental-cortical functions | 27.41±2.682 | 19-30 | -1.639 | 2.726 | 2.7 | 18.9 |
| PD-CRS total score | 87.30±11.244 | 61-109 | -0.107 | -0.226 | 2.7 | 2.7 |

PD-CRS: Parkinson’s Disease - Cognitive Rating Scale; SD: Standard deviation.

Supplement-Table2 Acceptability of PD-CRS in PD-MCI

| Item | Mean±SD | Min-Max | skewness | kurtosis | Floor effect (%) | Ceiling effect（%） |
| --- | --- | --- | --- | --- | --- | --- |
| Immediate free-recall verbal memory | 6.70±2.681 | 0-12 | -0.260 | -0.463 | 2.3 | 2.3 |
| Confrontation naming | 17.09±2.351 | 11-20 | -0.958 | 0.302 | 2.3 | 13.6 |
| Sustained attention | 5.32±2.785 | 0-10 | -0.135 | -0.880 | 4.6 | 4.6 |
| Working memory | 4.82±1.618 | 0-10 | 0.309 | 2.629 | 2.3 | 2.3 |
| Clock drawing | 7.82±2.026 | 2-10 | -1.023 | 0.543 | 2.3 | 20.5 |
| Copying a clock | 9.57±0.818 | 7-10 | -1.696 | 1.630 | 2.3 | 75 |
| Delayed free-recall verbal memory | 5.11±2.572 | 0-10 | -0.054 | -0.989 | 2.3 | 2.3 |
| Alternating verbal fluencies | 6.64±3.577 | 0-12 | -0.700 | -0.721 | 9.1 | 2.3 |
| Action verbal fluencies | 8.77±4.220 | 0-18 | 0.273 | -0.286 | 2.3 | 2.3 |
| Frontal-subcortical functions | 45.18±12.901 | 11-69 | -0.384 | -0.248 | 2.3 | 2.3 |
| Instrumental-cortical functions | 26.66±2.272 | 21-30 | -0.616 | 0.048 | 2.3 | 11.4 |
| PD-CRS total score | 71.84±14.144 | 34-99 | -0.274 | -0.129 | 2.3 | 2.3 |

PD-CRS: Parkinson’s Disease - Cognitive Rating Scale; SD: Standard deviation.

Supplement-Table3 Acceptability of PD-CRS in PDD

| Item | Mean±SD | Min-Max | skewness | kurtosis | Floor effect (%) | Ceiling effect（%） |
| --- | --- | --- | --- | --- | --- | --- |
| Immediate free-recall verbal memory | 4.73±1.191 | 4-7 | 1.507 | 0.877 | 63.7 | 18.2 |
| Confrontation naming | 14.91±2.809 | 10-18 | -0.813 | 0.066 | 18.2 | 27.3 |
| Sustained attention | 3.00±2.646 | 0-9 | 1.109 | 1.483 | 18.2 | 9.1 |
| Working memory | 3.64±1.362 | 2-6 | 0.230 | -0.967 | 27.3 | 9.1 |
| Clock drawing | 5.18±3.573 | 0-10 | -0.085 | -1.621 | 9.1 | 9.1 |
| Copying a clock | 6.73±3.495 | 0-10 | -1.181 | 0.095 | 9.1 | 18.2 |
| Delayed free-recall verbal memory | 2.36±2.420 | 0-6 | 0.204 | -1.840 | 45.5 | 9.1 |
| Alternating verbal fluencies | 3.55±3.045 | 0-10 | 0.920 | 0.579 | 18.2 | 9.1 |
| Action verbal fluencies | 4.82±3.219 | 0-11 | 0.618 | -0.026 | 9.1 | 9.1 |
| Frontal-subcortical functions | 27.27±11.577 | 11-48 | 0.611 | -0.676 | 9.1 | 9.1 |
| Instrumental-cortical functions | 21.64±4.523 | 15-28 | -0.170 | -1.653 | 9.1 | 9.1 |
| PD-CRS total score | 48.91±14.916 | 30-73 | 0.418 | -1.320 | 9.1 | 9.1 |

PD-CRS: Parkinson’s Disease - Cognitive Rating Scale; SD: Standard deviation.

Supplement-Table4 Cognitive function comparison between BDI<20 and ≥20 in PD-MCI

|  | BDI<20(n=37) | BDI≥20 (n=7) | p^a^ |
| --- | --- | --- | --- |
| Immediate free-recall verbal memory | 6.54±2.765 | 7.57±2.149 | 0.360 |
| Confrontation naming | 17.19±2.331 | 16.57±2.573 | 0.615 |
| Sustained attention | 5.19±2.904 | 6.00±2.082 | 0.469 |
| Working memory | 4.65±1.438 | 5.71±2.289 | 0.297 |
| Clock drawing | 7.76±2.140 | 8.14±1.345 | 0.950 |
| Copying a clock | 9.54±0.836 | 9.71±0.756 | 0.637 |
| Delayed free-recall verbal memory | 5.08±2.732 | 5.29±1.604 | 0.900 |
| Alternating verbal fluencies | 6.46±3.420 | 7.57±4.504 | 0.297 |
| Action verbal fluencies | 8.86±4.541 | 8.29±1.890 | 0.925 |
| Frontal-subcortical functions | 44.54±13.688 | 48.57±7.254 | 0.431 |
| Instrumental-cortical functions | 26.73±2.256 | 26.29±2.498 | 0.802 |
| PD-CRS total score | 71.27±14.968 | 74.86±8.764 | 0.509 |
| MDRS | 131.11±9.486 | 133.14±8.395 | 0.706 |

^a^Mann-Whitney U test

PD-CRS: Parkinson’s Disease - Cognitive Rating Scale; MDRS: Dementia rating scale

Supplement-Table5 Cognitive function comparison between BDI<20 and ≥20 in PDD

|  | BDI<20 (n=7) | BDI≥20 (n=4) | p^a^ |
| --- | --- | --- | --- |
| Immediate free-recall verbal memory | 4.57±1.134 | 5.00±1.414 | 0.509 |
| Confrontation naming | 15.71±1.604 | 13.50±4.123 | 0.557 |
| Sustained attention | 3.14±1.574 | 2.75±4.272 | 0.338 |
| Working memory | 3.57±1.512 | 3.75±1.258 | 0.772 |
| Clock drawing | 5.57±4.353 | 4.50±1.915 | 0.634 |
| Copying a clock | 6.00±4.282 | 8.00±0.816 | 0.924 |
| Delayed free-recall verbal memory | 2.43±2.370 | 2.25±2.872 | 0.921 |
| Alternating verbal fluencies | 3.14±2.340 | 4.25±4.349 | 0.771 |
| Action verbal fluencies | 4.57±3.952 | 5.25±1.708 | 0.446 |
| Frontal-subcortical functions | 27.00±11.387 | 27.75±13.672 | 1.000 |
| Instrumental-cortical functions | 21.71±5.090 | 21.50±4.041 | 0.848 |
| PD-CRS total score | 48.71±15.489 | 49.25±16.174 | 0.705 |
| MDRS | 115.29±19.155 | 112.50±9.327 | 0.449 |

^a^Mann-Whitney U test

PD-CRS: Parkinson’s Disease - Cognitive Rating Scale; MDRS: Dementia rating scale
