# Supplementary material for: Validation of Revised Chinese Version of PD-CRS in Parkinson's Disease Patients
Source: Parkinsons Dis. 2020 Feb 19;2020:5289136. doi: 10.1155/2020/5289136 (PMC7049872; doi:10.1155/2020/5289136)
Supplement: Supplementary Materials — Supplementary Table 1: acceptability of PD-CRS in NC; Supplementary Table 2: acceptability of PD-CRS in PD-MCI; Supplementary Table 3: acceptability of PD-CRS in PDD; Supplementary Table 4: cognitive function comparison between BDI <20 and ≥ 20 in PD-MCI; Supplementary Table 5: cognitive function comparison between BDI <20 and ≥ 20 in PDD. [file 5289136.f1.zip › PD-CRS scale.doc]

**帕金森病认知功能评分量表**

Parkinson’s Disease Cognitive Rating Scale (PD-CRS)

**1.即刻词语自由回忆**

指导语：受试者大声连续朗读12张卡片上的单词。读完后，让受试者尽可能多的回忆这些单词。该任务重复三次，每次结束都让受试者尽可能多的回忆单词。结束时，提醒受试者牢记这些单词，后面还需要回忆。

计分：每回忆起1个单词得1分，取三次任务中的最高分为最后得分（最低分=0，最高分=12）：

| **正确回忆的单词请打钩 (√)** | | | |
| --- | --- | --- | --- |
| **单词** | **第一次回忆：** | **第二次回忆:** | **第三次回忆:** |
| 灯光 |  |  |  |
| 丝绸 |  |  |  |
| 沙子 |  |  |  |
| 睫毛 |  |  |  |
| 大米 |  |  |  |
| 茶叶 |  |  |  |
| 黑板 |  |  |  |
| 自行车 |  |  |  |
| 星星 |  |  |  |
| 狮子 |  |  |  |
| 戒指 |  |  |  |
| 酱油 |  |  |  |
|  | 合计:______/12 | 合计:______/12 | 合计:______/12 |
| **总分（3次测试的最高得分）: ______/12** | | | |

**2.命名**

指导语：请受试者命名20张卡片中的线条图形，每次作答时间不限，但只给一次机会。不做任何提示。

计分：正确命名1次计1分（最低0分，最高20分）

| 回答正确的打钩 (√) | |
| --- | --- |
|  |  |
|  |  |
|  |  |
|  |  |
|  |  |
|  |  |
|  |  |
|  |  |
|  |  |
|  |  |
|  |  |
|  |  |
|  |  |
|  |  |
|  |  |
|  |  |
|  |  |
|  |  |
|  |  |
|  |  |
| **总分: ______/20** | |

**3.持续的注意力**

指导语：向受试者以每秒钟一个数字的速度朗读下列数字组合，让受试者回答其中有几个单数。共10个组合，分为5个逐渐增加难度的等级。测验开始时，先给受试者举例。当受试者连续答错两个组合时停止。

计分：答对1次计1分（最低0分，最高10分）

|  |  | **圈出正确的答案** |
| --- | --- | --- |
| 举例 | 574  836 | 2个单数  1 个单数 |
| 1. | 42731  75836 | 3个单数  3个单数 |
| 2. | 619473  392487 | 4个单数  3个单数 |
| 3. | 5917423  4179386 | 5个单数  4个单数 |
| 4. | 58192647  38295174 | 4个单数  5个单数 |
| 5. | 527184936  497361584 | 5个单数  5个单数 |
|  | **总分: ______/10** | |

**4.工作记忆力**

指导语：向受试者以每秒钟一个数字的速度朗读下列随机排列的数字组合。每读完一个组合，请受试者倒着背出数字。受试者连续答错两个组合时停止。

计分：答对1次计1分（最低0分，最高10分）：

|  |  | 圈出正确的答案 |
| --- | --- | --- |
| 举例 | 84  72 | 48  27 |
| 1 | 26  41 | 62  14 |
| 2 | 327  496 | 723  694 |
| 3 | 1528  6184 | 8251  4816 |
| 4 | 53941  72485 | 14935  58427 |
| 5 | 312936  473912 | 639213  219374 |
|  | **总分：______/10** | |

**画钟测试**

**5.自由画钟：**请受试者在空白纸上画一个钟面，指针指向10:25

计分：共分12项，每项符合要求计1分（最低0分，最高10分）

**6.临摹画钟：**请受试者临摹画钟

计分：共分12项，每项符合要求计1分（最低0分，最高10分）

12

11	1

10	2

9	**•**	3

8	4

7	5

**6**

|  | **自由画钟** | | **临摹复制画钟** | |
| --- | --- | --- | --- | --- |
| **正确完成打钩(√)** | **正确** | **错误** | **正确** | **错误** |
| 图形外观像钟表 |  |  |  |  |
| 无直线或扇形 |  |  |  |  |
| 数字标识对称 |  |  |  |  |
| 钟面上只有1-12这些数字 |  |  |  |  |
| 数字依次排序正确 |  |  |  |  |
| 画了两个指针：时针，分针 |  |  |  |  |
| 指针是带有箭头指示的 |  |  |  |  |
| 时针比分针短 |  |  |  |  |
| 只标记了数字（无字母） |  |  |  |  |
| 没有数字25 |  |  |  |  |
| **总分** | **______/10**  **自由画钟** | | **______/10**  **临摹复制画钟** | |

**7.延迟词语自由回忆**

请受试者回忆测验开始时的词语，只回忆一次。

计分：每个词语计1分（最低0分，最高10分）

| **单词** | **正确回忆的单词请打钩 (√)** |
| --- | --- |
| 灯光 |  |
| 丝绸 |  |
| 沙子 |  |
| 睫毛 |  |
| 大米 |  |
| 茶叶 |  |
| 黑板 |  |
| 自行车 |  |
| 星星 |  |
| 狮子 |  |
| 戒指 |  |
| 酱油 |  |
|  | 合计:______/12 |

**8. 交替词语流畅性**

请受试者在一分钟内尽可能多的交替说出衣物名称和以“ 发 ”开头的单词，两者交替进行，即：说一件衣物的名称，再说一个以“ 发 ”开头的单词，如此交替进行。

计分：每项完成正确计1分（最低0分，最高20分）

| **记录受试者给出的答案** | | | |
| --- | --- | --- | --- |
| **1.** | **2.** | **3.** | **4.** |
| **5.** | **6.** | **7.** | **8.** |
| **9..** | **10.** | **11.** | **12.** |
| **13.** | **14.** | **15.** | **16.** |
| **17.** | **18.** | **19.** | **20.** |
| **总分: ______/20** | | | |

**9.动作性语言流畅性**

请受试者在60秒内尽可能多的列举“人的动作”，仅说表示动作的词，不需要说句子。不能重复同一个动词，如“打球、打牌”则不行。

计分：每项完成正确计1分（最低0分，最高30分）

| **记录受试者给出的答案** | | | |
| --- | --- | --- | --- |
| **1.** | **2.** | **3.** | **4.** |
| **5.** | **6.** | **7.** | **8.** |
| **9..** | **10.** | **11.** | **12.** |
| **13.** | **14.** | **15.** | **16.** |
| **17.** | **18.** | **19.** | **20.** |
| **21.** | **22.** | **23.** | **24.** |
| **25.** | **26.** | **27.** | **28.** |
| **29..** | **30.** |  |  |
| **总分: ______/30** | | | |

**总分表**

| 项目 | 得分 |
| --- | --- |
| 1. 即刻词语自由回忆 | ________/12 |
| 2. 命名 | ________/20 |
| 3. 持续的注意力 | ________/10 |
| 4. 工作记忆力 | ________/10 |
| 5. 自由画钟 | ________/10 |
| 6. 临摹画钟 | ________/10 |
| 7. 延迟词语自由回忆 | ________/12 |
| 8. 词语流畅性 | ________/20 |
| 9. 动作性语言流畅性 | ________/30 |
| 额叶-皮质下功能区评分 | ________/104 |
| 后皮质功能区评分 | ________/30 |
| 总分 (0-134) | ________/134 |
